# Toxicity of combined exposure to acrylamide and *Staphylococcus aureus*

**DOI:** 10.1016/j.toxrep.2022.04.018

**Published:** 2022-04-20

**Authors:** Yuko Shimamura, Takuya Yui, Hayao Horiike, Shuichi Masuda

**Affiliations:** School of Food and Nutritional Sciences, University of Shizuoka, 52-1 Yada, Suruga-ku, Shizuoka 422-8526, Japan

**Keywords:** BHI, Brain heart infusion, FBS, Fetal bovine serum, *Staphylococcus aureus*, Virulence factors, Epoxy compounds, Comet assay, DNA damage, Bovine serum albumin

## Abstract

In the current study, the aim was to examine the toxicity of combined exposure to acrylamide and *Staphylococcus aureus*. We investigated the effect of staphylococcal enterotoxin A (SEA), a toxin produced by *Staphylococcus aureus,* on the oxidation induced DNA damaging potency of acrylamide in mouse spleen cells using an Formamidopyrimidine-DNA glycosylase-modified (FPG)-modified comet assay. Parameters like tail moment, tail length, and % tail DNA as indicators of DNA damage were significantly increased in the combined acrylamide and SEA treatment compared with SEA or acrylamide alone. Further, we examined the effects of acrylamide and its epoxide metabolite glycidamide on overall production of SEA, SEA mRNA gene expression, and on the formation of biofilm of *S. aureus*. Acrylamide significantly increased the SEA expression level and SEA production in *S. aureus*. Acrylamide also significantly increased biofilm formation in *S. aureus* without affecting its growth rate. Moreover, the addition of acrylamide significantly increased the expression of *S. aureus* virulence factors RNAIII and *ica*A in fetal bovine serum. Our results showed that combined exposure to acrylamide and *S. aureus* or its toxin enhanced their chemical and biological toxicities.

## Highlights

• The combined exposure of acrylamide and bacterial toxins enhanced the genotoxicity of AA.
• Acrylamide enhanced the expression and production of staphylococcal enterotoxin A.
• Acrylamide enhanced the biofilm formation of *S. aureus.*

## Graphical Abstract

**Figure:**
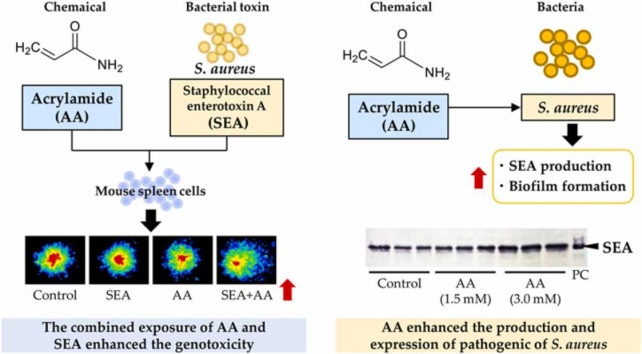

## 1 Introduction

Exposure to chemical risk factors, such as toxic chemicals, and biological risk factors, such as pathogenic bacteria, in food environments pose health risks. Hazardous chemical exposure can generate genetic abnormalities, which cause diseases, such as cancer and immune dysfunction [1]. In addition, pathogenic bacteria, such as *Staphylococcus aureus*, can cause several severe diseases, including food poisoning [2], skin infections, and respiratory tract infections [3]. Although we are routinely exposed to both of these risk factors in food environments, assessments of combined exposure toxicity are limited.

Acrylamide is a common organic chemical in food that forms when hydrocarbons are subjected to high temperatures [4]. High acrylamide concentrations are often found in food products, including coffee, fried potatoes and bread [5]. Acrylamide is metabolized in vivo to the more reactive epoxide glycidamide [6]. Acrylamide and glycidamide are strongly genotoxic substances that have been shown to damage DNA and form adducts with DNA and proteins [7], [8], bonding to protein thiol groups and nucleic acid amino groups. Acrylamide has been classified as a probable human carcinogen under Group 2 A by the International Agency for Research on Cancer (IARC 1994). Although many mechanisms of acrylamide toxicity are well-recognized, some aspects of toxicity remain unknown [9]. Average acrylamide consumption in foods is generally much lower than the doses tested in animal studies. Moreover, evaluations of combined exposures to acrylamide and other common toxins in foods are limited.

Staphylococcal enterotoxin A (SEA), produced by *S. aureus*, is a common bacterial toxin in foods and the leading causative agent of food poisoning in humans [10], [11], [12], [13]. SEA is a super antigen, meaning it can stimulate excessive immune system activity and subsequently induce diseases [14], [15]. Super antigens of the bacterial toxins bind to MHC class II molecules causing a large proportion of T cells to get activated via direct binding with the T cell receptor [16], [17]. The *S. aureus* accessory gene regulator (*agr*), a global regulator is constituted of two main transcripts, designated RNAII and RNAIII. The *agr* gene is known to modulate the expression of multiple exoproteins, such as staphylococcal enterotoxins B, C & D, alpha-hemolysin, protein A and toxic shock syndrome toxin 1[18]. Conversely, the expression of SEA has not been seen to get affected by *agr* expression [19]. *S. aureus* is known to form biofilms in host tissues, which requires *agr* repression [20].

It has been reported that flumequine, a fluoroquinolone antibiotic, enhanced 2-amino-3, 8-dimethylimidazo [4, 5-f] quinoxaline (MeIQx)-induced mutagenicity in animal models [21]. Like flumequine, SEA enhances cell proliferation and cytokine secretion [22], suggesting that combined exposure to toxic chemicals and SEA may induce mutagenicity. SEA and acrylamide are both found in foods and, thus, could be simultaneously ingested by humans, resulting in co-exposure to these toxins. SEA and acrylamide may produce additive or synergistic toxic effects, which have yet to be evaluated.

The objective in the current study was to examine the effects of SEA on DNA damage in mouse spleen cells caused by acrylamide using a formamidopyrimidine-DNA glycosylase (Fpg)-modified comet assay. Additionally, the effects of acrylamide on the expression of virulence factors, of *S. aureus* were also assessed for expression including the SEA.

## 2 Material and methods

### 2.1 Chemicals

Acrylamide and glycidamide (Wako Pure Chemical Industries, Ltd. (Osaka, Japan). Fpg protein from *Escherichia coli* (Sigma-Aldrich Japan (Tokyo, Japan)). Staphylococcal enterotoxin A (SEA; purity >95%) (Toxin Technology (Sarasota, FL, USA)) were used.

### 2.2 Isolation of mouse spleen cells

Mouse spleen cells were obtained from C57BL/6 J female mice (nine weeks) as described previously [23]. Animal experimental procedures were approved by the Institutional Animal Care and Use Committees of the University of Shizuoka (Permit Numbers: 185185 and 195220). Spleen cells (1 × 10^7^ cell/mL) were placed in a 30-mm Petri dish in 1.96 mL of Russ-10 medium with 20 μL of 10 mM acrylamide (final concentration 0.1 mM, with Fpg treatment) or 1.5 M acrylamide (final concentration 15 mM, without Fpg treatment), with and without 20 μL of 5 μg/mL SEA (50 ng/mL). Milli-Q water was used as a control. All treatments were incubated at 37 °C in a 5% CO_2_ incubator for 16 h. After the incubation, the reaction mixture was centrifuged at 1500 × *g* for 5 min at 4 °C, and the supernatants were resuspended in 0.1 mL PBS.

### 2.3 Comet assay and Fpg-modified comet assay

The Comet assay was performed in accordance with OECD test guideline 489. Glass slides were pretreated with 0.7% agarose solution (normal melting point) over the surface of slide. A 75 μL aliquot of reacted mouse spleen cells was mixed with 75 μL of 1.4% agarose solution (low melting point) and placed on pretreated glass slide. The slide was covered with a cover glass, and refrigerated for at least 30 min until agarose gel was fixed. following which the cover glass was removed and immersed into 150 mL of cold lysing solution (2 M NaOH, 2.5 M NaCl, 100 mM EDTA-2 Na, 10 mM Tris, 1.1% Sodium *N*-lauroyl sarcosinate, 1% TritonX100, 10% DMSO, pH 10) and kept overnight at 4 °C. In the case of Fpg treatment, the cell sample after cell lysing was immersed in buffer for Fpg treatment (100 mM NaCl, 0.01% BSA, 12.5 mM tris-buffer, 1 mM EDTA), which was changed every 5 min thrice. 80 μL of the Fpg containing solution was dropped onto the gel on slide, covered with a cover glass and slide placed in an incubator at 37 °C for 15 min (Fpg;1 μg/mL in buffer was used as control). The Fpg-treated and non-treated slides were placed in electrophoresis device for comet assay, submersed in alkali electrophoresis buffer (0.3 M NaOH, 1 mM EDTA-2Na, pH13, 4 °C) for 20 min. Electrophoresis was performed at 25 V, 0.3 A for 20 min at 4 °C. After electrophoresis, the slide was immersed in a neutralization buffer (0.4 M tris-buffer; pH7.5) for 20 min and then dehydrated by soaking with absolute ethanol (99.6%) for 10 min. Final staining was done with 20 µg/mL ethidium bromide before image analysis. Image analysis were performed using a fluorescence microscope (Nikon, Tokyo, Japan) equipped with an image analysis software (Comet Analyzer, YOU WORKS, Tokyo, Japan).

To assess the DNA damage degree, the percentage of DNA in the tail was measured in approximately 100 cells per slide (200 × using software Comet Assay IV (Perceptive Instruments).

### 2.4 Bacterial strain

This study utilized the SEA-producing strain *S. aureus* C-29 [24], which was isolated from human hands [25]. The strain was grown in a brain heart infusion (BHI; Difco Laboratories, Detroit, MI, USA) broth at 37 °C.

### 2.5 Influence of acrylamide on the growth of S. aureus

Acrylamide and glycidamide were dissolved in Milli-Q water and passed through a sterilized membrane filter (0.2-μm cellulose nitrate) *S. aureus* C-29 stock culture was inoculated into BHI broth at 37 °C for 18 h while undergoing constant shaking. After incubation, 10 μL of *S. aureus* C-29 culture fluid (~10^8^ CFU mL) and acrylamide solution (at 1.5, 3.0, 4.5, 6.5, 7.5, and 15 mM concentrations) or glycidamide solution (1.5, 3.0, and 4.5 mM concentrations) were added to BHI broth (180 μL) followed by incubation at room temperature for 24 h. *S. aureus* C-29 growth was assessed by optical density measurement at 660 nm.

### 2.6 SDS-PAGE & Western blot Assays

The amount of SEA production of *S. aureus* C-29 cultured in fetal bovine serum (FBS) containing acrylamide (AA) was examined. The Western blot analysis was previously described in detail [24]. Separation of the resultant supernatants was performed on 15% SDS-PAGE and transferred to a polyvinylidene difluoride membrane. The membranes were blocked in phosphate-buffered saline containing 0.05% Tween 20 and blocked with 0.6% skim milk for 2 h and incubated at 4 °C overnight with rabbit anti-SEA IgG (Sigma, St. Louis, Mo., USA), followed by incubation for 1 h with goat anti-rabbit peroxidase (KPL). The immunoreactive bands were detected using a BCIP/NBT substrate (KPL) and quantified using Image J software (Natl. Inst. of Health, Bethesda, Md., USA).

### 2.7 Crystal violet assay

*S. aureus* C-29 stock culture was inoculated into BHI broth at 37 °C for 18 h while undergoing constant shaking. After incubation, 20 μL of *S. aureus* culture (~10^8^–10^9^ CFU mL) and acrylamide or glycidamide solution (3.0 mM final concentration) was added to 100 μL of 66% tryptic soy broth (Nissui Pharmaceutical Co., Ltd., Tokyo, Japan) containing 0.2% glucose in 96-well tissue culture plates and incubated for 48 h at 37 °C. Planktonic bacteria were cleansed from the wells via sterile distilled water and then 125 μL 0.1% crystal violet solution was added and incubated for 10 min at room temperature. Excessive crystal violet stains were eliminated from the wells by rinsing with sterile distilled water three times. The wells were air-dried for 10 min at room temperature before 100 μL of 99.5% ethanol was added for 10 min for de-staining. The quantification of biofilm formation was done by absorbance measurement at 595 nm by a microplate reader. The relative biofilm formation was calculated as below (Eq. 1):

(1) $$\mathrm{Relative biofilm formation}=\left\{\frac{\mathrm{mean}{\mathrm{OD}}_{595}\mathrm{of treated well}}{\mathrm{mean}{\mathrm{OD}}_{595}\mathrm{of control well}}\right\}\times 100$$

### 2.8 RNA isolation & real-time PCR

*S. aureus* C-29 stock culture was inoculated into BHI broth at 37 °C for 18 h while undergoing constant shaking. A 30 μL aliquot of *S. aureus* C-29 culture fluid (~10^8^–10^9^ CFU mL) and 30 μL of acrylamide or glycidamide solution (1.5 and 3.0 mM concentrations respectively) were added to BHI broth (3 mL) followed by RT incubated for 4 h. Furthermore, to better replicate in vivo conditions, FBS (Gibco Life Technologies Japan) was used instead of BHI broth. Prior to use, FBS was heated to 56 °C for 30 min *S. aureus* cells grown at 37 °C for 18 h were harvested and washed with 6% Chelex-100 solution, suspended in FBS, and the optical density (660 nm) was adjusted to 1.0. The bacterial suspension (30 μL) was inoculated into 3 mL of FBS or BHI broth, then 30 μL of acrylamide solution (final concentrations of 1.5 and 3.0 mM) was added and the mixture was incubated at 37 °C for 24 h. After incubation, each bacterial cell was collected using centrifugation (14,000 g, 5 min).

RNA extraction was done using a RiboPure-Bacteria kit (Ambion, Austin, TX, USA) in accordance with the manufacturer's instructions. Real-time PCR was performed using SYBR Premix Ex Taq (Takara, Shiga, Japan) and a real-time PCR system (Thermal Cycler Dice® Real-Time System Single; Takara). Each sample was normalized to 16 S rRNA, triplicates were averaged, and relative mRNA levels were determined. Table S1 has details of the primers used for RT-PCR (supplementary data).

### 2.9 Statistical analysis

Results were compared by Student’s *t*-test or one-way ANOVA, followed by Dunnett’s test using Microsoft Excel 2016 (Microsoft, Redmond, WA, USA). The significance level was set at *p* < 0.05, with each experiment having at least three replicate values.

## 3 Results and discussion

### 3.1 Effect of S. aureus virulence factors on AA-induced DNA damage

**Fig. 1:**
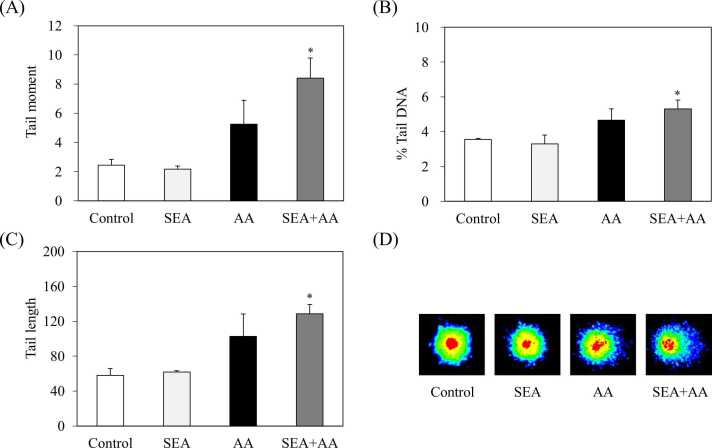
Mouse spleen cell DNA damage induced by exposure to staphylococcal enterotoxin A (SEA) and acrylamide (AA). Comet assay results, showing tail moment (A), tail length (B), % tail DNA (C), and representative microscopic images (D). Error bars show standard error. * indicates *p* < 0.01 compared to the control (sterile water) (*n* = 3).

Mouse spleen cell DNA damage with combined exposure to acrylamide (15 mM) and SEA (50 ng/mL) was evaluated using the comet assay. When SEC and SEL (100 μg), which belong to the same SEs family as SEA, were orally administered to the mice, the maximum serum concentration were 81.1 ± 16.2 and 35.9 ± 4.8 ng/mL, respectively [26]. In a previous study, we also reported that exposure of mouse spleen cells to 50 ng/mL of SEA significantly increased the mRNA expression level of IFN-γ [27]. Therefore, 50 ng/mL SEA, which shows immunotoxicity (no genotoxicity), was used in this study. Fig. 1 showed tail moment (Fig. 1A), tail length (Fig. 1B), and % tail DNA (Fig. 1C) calculated automatically from the images of nuclear intensity and tail intensity of individual cells (Fig. 1D). No changes in tail moment, tail length, and % tail DNA were observed after exposure to SEA alone (Fig. 1). However, in spleen cells co-exposed to acrylamide and SEA, tail moment (Fig. 1A), tail length (Fig. 1B), and % tail DNA (Fig. 1C) were increased significantly in comparison to the used control (Milli-Q water) (*p* < 0.05).

**Fig. 2:**
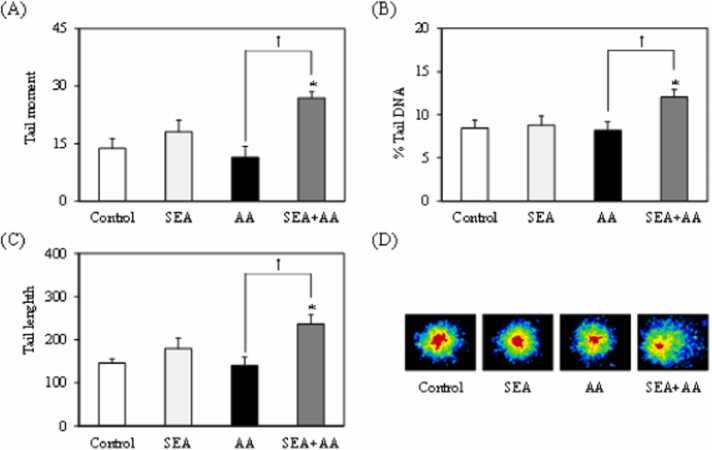
Mouse spleen cell oxidative DNA damage induced by exposure to staphylococcal enterotoxin A (SEA) and acrylamide (AA). (A)FPG comet assay results, showing tail moment, (B) tail length percentage, (C) tail DNA and representative microscopic images (D). Error bars show standard error. * indicates *p* < 0.01 compared to the control (sterile water). † indicates *p* < 0.01; un*p*aired, two-tailed Student’s *t*-test (*n* = 3).

In order to further clarify effect of the combined exposure to acrylamide and SEA, DNA damage was evaluated using an Fpg-modified comet assay, which increases the detection sensitivity by approximately 2.5 times. Tail moment, tail length, and % tail DNA were significantly increased in spleen cells exposed to acrylamide and SEA compared with the control and acrylamide alone (Fig. 2) (*p* < 0.05). These results suggest that combined exposure to chemicals and bacterial toxins may increase their genotoxicity.

DNA damage has the potential to induce cell death and mutations, which may result in genetic diseases, carcinogenesis, aging, and ultimately, death [28]. The combined acrylamide and SEA exposure exacerbates DNA damage can lead to subsequent inducing of strand breaks in DNA, which changes the quality of the damage. DNA damage can include not only DNA strand breaks but also DNA base damage. It has been demonstrated that SEA stimulates the proliferation of T cell in spleen cells and then subsequent cytokine production in C57BL/6 female mice [29]. Jaiswal et al. previously demonstrated that the inflammatory cytokines cause induction of DNA damage and subsequently inhibit the DNA repair [30], and these effects could be attributed to the compounded effects of SEA and acrylamide on DNA. Hence, the type of DNA damage produced by the co-exposure of acrylamide and SEA requires further study.

### 3.2 Effect of acrylamide and glycidamide on SEA expression and production

**Fig. 3:**
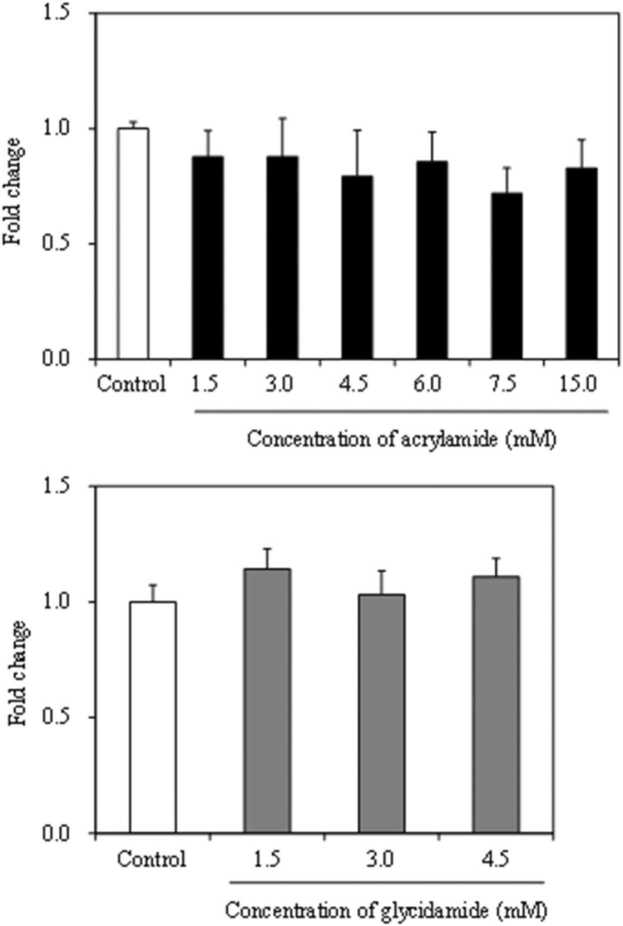
Effects of acrylamide and glycidamide on *Staphylococcus aureus* growth. Error bars show standard deviation (*n* = 3).

We first examined the effects of acrylamide and glycidamide on *S. aureus* growth and conducted tests with maximum concentrations of acrylamide and glycidamide. In a previous study, micronucleus tests using human lymphoblastoid TK6 cells showed positive results at 15.0 mM acrylamide (−S9) and 1.5 mM glycidamide (+/−S9) [31]. Since 1.5 mM glycidamide did not affect SEA mRNA expression in *S. aureus*, the concentration of glycidamide was increased. When acrylamide was administered intraperitoneally to SD rats at 5 or 100 mg/kg body weight, 51% and 13% (average about 30%) of the administered acrylamide were metabolized to glycidamide, respectively [32]. Considering the result and report, maximum concentrations of acrylamide and glycidamide were set at 15.0 mM and 4.5 mM (assuming 30% of 15.0 mM acrylamide was metabolized to glycidamide), respectively. No significant effect on *S. aureus* growth (Fig. 3) was observed at the concentrations tested. Therefore, all the subsequent assays were done at non-growth inhibiting concentrations.

**Fig. 4:**
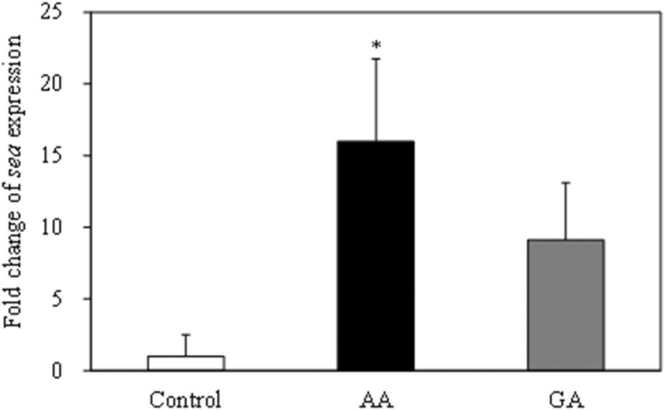
Effect of acrylamide (AA) and glycidamide (GA) on SEA relative mRNA expression in *S. aureus*. Error bars show standard error (*n* = 3). * indicates *p* < 0.05 compared to the control.

**Fig. 5:**
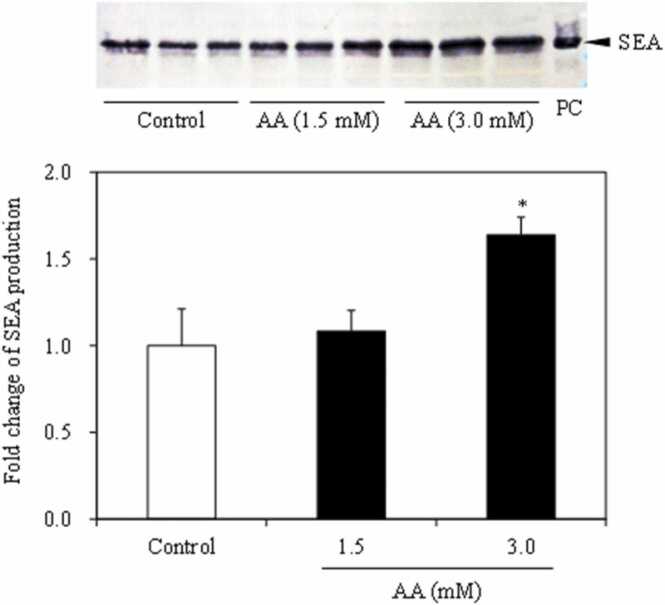
Effect of acrylamide (AA) and glycidamide (GA) on the relative production of staphylococcal enterotoxin A (SEA) in *Staphylococcus aureus.* SEA production was determined using Western blot assay with anti-SEA antibodies; fold-change was determined relative to the control (sterile water). Error bars show standard error (*n* = 3). * indicates *p* < 0.05 compared to the control.

The effects of acrylamide and glycidamide on *S. aureus* SEA mRNA expression levels were examined. The SEA gene expression level was 9.1% higher in the glycidamide treatment compared to the control; however, the difference was not statistically significant (Fig. 4). Acrylamide significantly increased (16.0%) the SEA expression level (*p* < 0.05) (Fig. 4). We then examined the SEA protein production level in *S. aureus* exposed to acrylamide. The amount of SEA produced significantly increased with exposure to acrylamide at concentrations of 3 mM (*p* < 0.05) (Fig. 5).

In *S. aureus*, it has been observed that the oxidative stress or induced cell damage [33] activates the SOS response system, inducing prophage [34] and subsequently upregulating SEA expression. In bacteria, the SOS response system is activated by DNA damage or delayed DNA replication which in turn may be caused by exposure to stress conditions [35], like antibiotic treatment [36], starvation [37], and oxidative stress [38]. The SOS response can also be activated by exposure to hostile environments during food processing, such as high NaCl concentrations (≥2%), heat, UV irradiation, and low pH [39], [40], [41]. A previous study reported that acetic acid, which is a common food ingredient, upregulated the gene expression of the phage-encoded *SEA* in *S. aureus* by lowering the intracellular pH [42]. Repairing damaged DNA is essential for bacterial survival but can introduce mutations into the genome [43], [44], potentially increasing virulence factor expression in *S. aureus*. Moreover, acrylamide and glycidamide can form adducts with protein, DNA, and hemoglobin [45] and may induce the bacterial DNA damage response.

Additionally, acrylamide may also act as a nitrogen source. The optimum pH for SEB production by *S. aureus* was 7.0–7.5 I presence of a nitrogen source in comparison to 8.0 − 8.5 pH when there is a lack of a nitrogen source [46]. Further investigation is needed as some chemicals in foods can lead to the induction of prophage and, therefore, high SEA levels.

### 3.3 Effect of acrylamide and glycidamide on S. aureus biofilm formation

**Fig. 6:**
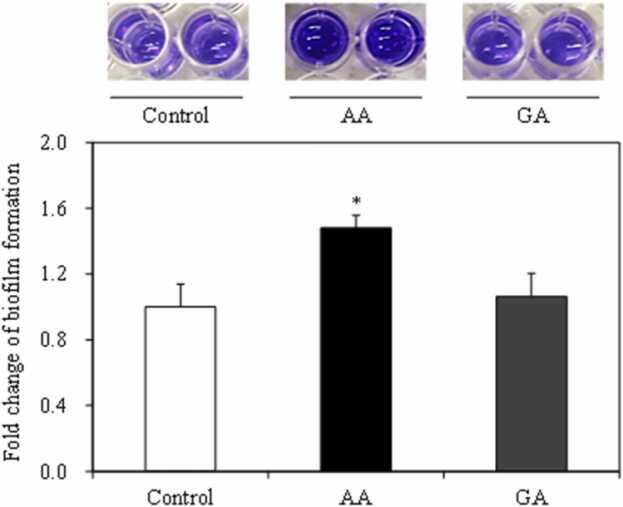
Effect of acrylamide (AA) and glycidamide (GA) on biofilm formation in *Staphylococcus aureus*. Biofilm formation was measured via crystal violet at absorbance 595 nm (top image). Error bars show standard error (*n* = 6). * indicates *p* < 0.05 compared to the control.

The effects of acrylamide and glycidamide on formation of staphylococcal biofilm were evaluated. Glycidamide did not affect the formation of biofilm, significantly. However, acrylamide significantly increased biofilm formation at a concentration that did not affect *S. aureus* growth (*p* < 0.05) (Fig. 6). These results suggest that acrylamide does not stimulate biofilm formation by accelerating growth.

Bacteria form biofilms in many habitats, either natural or experimental, attaching to inanimate or aquatic surfaces [47]. *S. aureus* biofilm formation can be induced oxidative stress or DNA damage, caused by exposure to materials, such as heparin [48] and cigarette smoke [49]. Activation of the SOS response system by antimicrobials and DNA damaging agents induces biofilm formation in several species of bacteria [50]. Acrylamide may trigger the SOS response system and, thus, induce biofilm formation. In addition, it has been reported that strains with amidase, an acrylamide-degrading enzyme, may be able to use acrylamide as a carbon source [51]. *S. aureus* produces several amidases [52], which may have promoted biofilm formation by capitalizing on acrylamide as a carbon and nitrogen source. On the other hand, glycidamide, which had no effect on biofilm formation, and on the expression of SEA mRNA described previously, may have reacted with the medium components before acting on *S. aureus*, given its higher reactivity compared to acrylamide. In the future, the effects of acrylamide and glycidamide on *S. aureus* should be examined in consideration of in vivo conditions.

Changes in osmotic pressure in culture media have also been reported to affect *S. aureus* biofilm formation. This phenomenon is associated with the altered expression of genes that contribute to the modulation of biofilm formation, including intercellular adhesion (*ica*) [53]. The production of biofilm is dependent on the presence of the *icaADBC* gene cluster (intracellular adhesion locus). A previous study showed that NaCl exposure not only induced SOS response but also reacted with *ica*A to increase biofilm formation [54]. Therefore, we examined the effect of acrylamide on the expression of factors responsible for virulence, like the *ica*A.

### 3.4 Effect of acrylamide on the virulence factors of S. aureus in FBS

*S. aureus* growth conditions are significantly different in the host and in the medium, and this can be due to the changes in the expression of virulence factors in the host. Therefore, we examined the effect of acrylamide on the expression of the virulence factors in *S. aureus* which was grown in FBS. RNA III is considered to be a global regulator for expression of many virulence factors [55]; hence, we targeted its regulation for observation. For biofilm formation, the main global regulators and the accessory gene regulator like *agr* quorum-sensing system, both are involved [50]. The *agrBDCA* operon comprises of two units P2 and P3, and these units encode the RNAII and RNAIII transcripts. As an *RNAII acts as the* internal effector molecule, and regulates the expression of agr-controlled virulence factors [55].

**Fig. 7:**
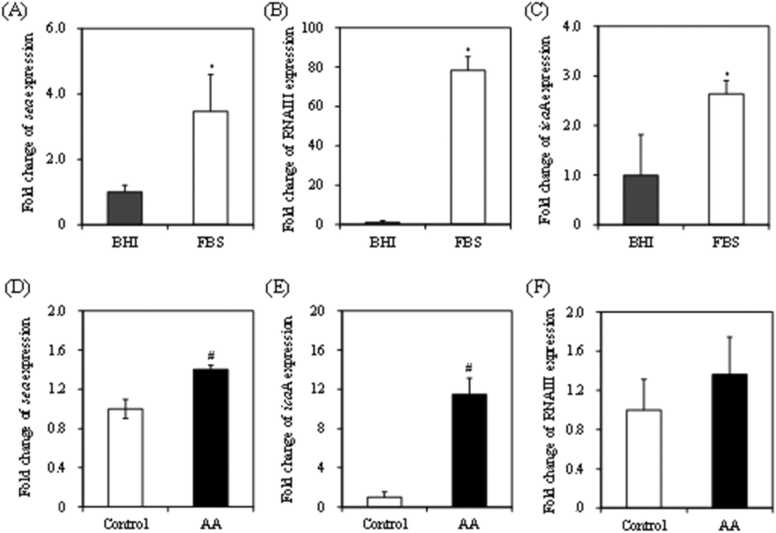
Comparison of relative SEA expression in *S. aureus* in BHI broth (A–C) or FBS (D–F). Error bars show standard error (*n* = 3). * indicates *p* < 0.05 compared to BHI, # indicates *p* < 0.05 com*p*ared to the control.

As a result, the levels of expression for the virulence factors, SEA (Fig. 7A), RNAIII (Fig. 7B), and *ica*A (Fig. 7C) genes were upregulated significantly in FBS compared to BHI medium (*p* < 0.05). RNAIII expression in FBS was almost 80-fold higher than that in BHI medium (Fig. 7B). This result is consistent with Oogai et al. [56], who reported that *S. aureus* RNA III expression was 10- to 100-fold higher in calf serum than in trypticase soy broth.

We then compared *S. aureus* virulence factor expression in FBS with acrylamide and without. SEA (Fig. 7D) and *ica*A (Fig. 7E) expressions were significantly higher in FBS with acrylamide (*p* < 0.05); *ica*A expression increased almost 10-fold (Fig. 7E). RNAIII tended to increase (Fig. 7F), but the difference was not significant. In addition, the levels of SEA expression were different in FBS (Fig. 7D) and BHI (Fig. 4). The above results therefore, are suggestive that the patterns of expression of *S. aureus* and acrylamide virulence factors may differ in vivo.

Taken together, our results show that combined exposure to acrylamide and *S. aureus* or its toxin affected their chemical and biological toxicities. The DNA damaging potency and mutagenicity of acrylamide and the expression of pathogenic factors, like production of toxin and formation of, biofilm in *S. aureus* were enhanced when combined. Our findings highlight the importance of combined exposure studies for chemicals and pathogenic bacteria in food environments.

## CRediT authorship contribution statement

**Yuko Shimamura:** Conceptualization, Methodology, Formal analysis, and Writing – original draft. **Takuya Yui:** Investigation, Visualization. **Hayao Horiike:** Validation, Investigation, Visualization. **Shuichi Masuda:** Writing – review & editing, Project administration, Funding acquisition.

## Declaration of Competing Interest

No potential conflicts of interest were disclosed.
