# Supplementary material for: Toxicity of combined exposure to acrylamide and Staphylococcus aureus
Source: Toxicol Rep. 2022 Apr 20;9:876–82. doi: 10.1016/j.toxrep.2022.04.018 (PMC9743448; doi:10.1016/j.toxrep.2022.04.018)
Supplement: Supplementary file 1 — Supplementary material [file mmc1.pdf]

Table S1. Primers for real-time RT-PCR used in this study

Gene name	Specific primers for real-time RT-PCR	
	Forward	Reverse
16S rRNA	gcg aag aac ctt acc aaa tc	cca aca tct cac gac acg
sea	aaa ata cag tac ctt tgg aaa cgg tt	ttt cct gta aat aac gtc ttg ctt ga
RNAIII	cga tgt tgt tta cga tag ctt	cca tcc caa ctt aat aac ca
icaA	agt tgt cga cgt tgg cta	cca aag acc tcc caa tgt
